# Nuclear Interaction between ADR-Induced p65 and p53 Mediates Cardiac Injury in iNOS (−/−) Mice

**DOI:** 10.1371/journal.pone.0089251

**Published:** 2014-02-25

**Authors:** Marsha P. Cole, Jitbanjong Tangpong, Terry D. Oberley, Luksana Chaiswing, Kinsley K. Kiningham, Daret K. St. Clair

**Affiliations:** 1 Biochemistry and Molecular Biology, University of Louisville, Louisville, Kentucky, United States of America; 2 School of Allied Health Sciences and Public Health, Walailak University, Nakhon Si Thammarat, Thailand; 3 Department of Pathology, VA Hospital, University of Wisconsin, Madison, Wisconsin, United States of America; 4 Pharmaceutical, Social and Administrative Sciences, Belmont College of Pharmacy, Nashville, Tennessee, United States of America; 5 Graduate Centers for Toxicology and Nutritional Sciences, University of Kentucky, Lexington, Kentucky, United States of America; National Institutes of Health, United States of America

## Abstract

Adriamycin (ADR) treatment causes an imbalance in the levels of nitric oxide (^•^NO) and superoxide (O_2_^•−^) production leading to cardiac injury. Previously we demonstrated that mice lacking inducible nitric oxide synthase (iNOS) have increased oxidative stress and mitochondrial injury. The molecular events leading to increased mitochondrial injury in iNOS deficient mice is unknown. ADR in the absence of iNOS preferentially activates a proapoptotic pathway without a concurrent increase in prosurvival pathways. Treatment with ADR leads to an increase in DNA binding activity of nuclear factor kappa B (NFκB) and p53 in wildtype mice. Following ADR treatment, p53, but not NFκB DNA binding activity, as well as the level of Bax, a p53 target gene, was increased in iNOS (−/−) mice. This apoptotic signaling effect in iNOS (−/−) is alleviated by overexpression of manganese superoxide dismutase (MnSOD). Increases in NFκB and p53 in ADR-treated wildtype mice did not lead to increases in target genes such as MnSOD, bcl-xL, or Bax. Moreover, co-immunoprecipitation analysis revealed that p65, a prominent member of the NFκB family, interacts with p53 in the nucleus. These results suggest that NFκB and p53 may counter act one another's actions in ADR-treated wildtype (WT) mice. Further, these results identify a novel mechanism by which oxidative stress may regulate transcription of proapoptotic genes.

## Introduction

Adriamycin (ADR) remains one of the most widely used chemotherapeutic agents, although there is a known dose-dependent cardiac toxicity. The cardiac injury associated with the use of ADR is hypothesized to be related to subsequent increases in superoxide (O_2_^•−^) production. The quinone containing anthracycline participates in a one electron reduction reaction in which molecular oxygen accepts an electron, thus producing O_2_^•−^. Redox cycling of ADR occurs through both enzymatic and non-enzymatic reactions. Enzymes such as cytochrome P-450 reductase, NADH dehydrogenase, xanthine oxidase, as well as non-enzymatic reduction catalyzed by iron, have been shown to participate in the redox cycling of ADR and the formation of O_2_^•−^ [1].

Adriamycin-induced cardiac toxicity has been proposed to be a consequence of mitochondrial injury [2]. Ultrastructural analysis of heart tissue following treatment with ADR reveals mitochondrial swelling, loss of cristae, and vacuolization. Previously, we have demonstrated that overexpression of manganese superoxide dismutase (MnSOD) is protective against ADR-induced cardiac injury using a transgenic mouse model [2]–[4]. This result suggests that O_2_^•−^ production as a result of ADR treatment most likely occurs in the mitochondria. Additionally, inducers of MnSOD protein and activity such as phenylbutyrate, a histone deacetylase inhibitor, are also cardioprotective in ADR-induced toxicity [5].

The role of nitric oxide (^•^NO) in ADR-induced cardiac injury is not well established. Inducible nitric oxide synthase (iNOS) null mice exhibited an exacerbation of injury in response to ADR treatment as compared to wildtype (WT) mice identified by ultrastructural analysis, biochemical markers such as serum creatine phosphokinase (CPK), lactate dehydrogenase (LDH), and cardiac troponin (cTnI) [6]. In addition, levels of serum nitrate and nitrotyrosine adducted proteins increased in WT mice following ADR treatment, but were absent in iNOS (−/−) mice [2]–[4]. Although these results provide insight leading to potential explanations for the exacerbation of injury in iNOS null mice, the mechanism is not clear.

In the present study, we utilize an acute model of ADR toxicity to further elucidate the possible mechanism(s) by which ROS and ^•^NO may serve as signal mediators regulating expression of anti- and apoptotic target genes. We demonstrate that activation of NFκB and p53 transcription factors interact to offset the expression of cytoprotective (MnSOD and bcl-xL) and proapoptotic (Bax) genes.

## Materials and Methods

### Generation of mice

Inducible NOS knock-out mice were purchased from Jackson Laboratories (Bar Harbor, ME) in the C57BL/6 background. The iNOS (−/−) mice were bred into the B6C3 background for greater than 10 generations and the colony was maintained at the University of Kentucky. Three lines of MnSOD over-expressing mice were generated in the B6C3 background using human MnSOD cDNA [2]. The human β-actin 5′ flanking sequence and promoter was used to target mRNA expression predominantly in the heart tissue [2]. The medium expressing line (TgM (+/−)) was bred to obtain homozygous MnSOD overexpression (TgM (+/+)). The iNOS (−/−) - TgM (+/+) cross was generated by sequential selection and back-crossing between iNOS (−/−) and TgM (+/+) mice [6]. Homozygosity was confirmed by back-crossing to WT mice. After more than 10 generations of back-crossing, male mice, 8–10 weeks old were used for experiments. Age- and gender-matched WT mice were bred and maintained in the B6C3 background to serve as controls. Procedures that involved mice were approved by the University of Kentucky Animal Care and Use Committee and were conducted in accordance to all policies for the use and care of laboratory research animals as set-forth by the NIH.

### Southern blot analysis

Genomic DNA was isolated from mouse tail as previously described [7]. The DNA was digested overnight at 37°C with *Bam*H I or *Pst* I, and separated on 0.7% or 0.9% agarose. The gel was denatured and neutralized prior to transferring overnight to a Nytran membrane (Schleicher & Schuell, Keene, NH). Using a vacuum oven pre-warmed to 80°C, the blot was baked for 2 h. Prior to hybridization with probe, the blot was prehybridized for 5 h at 42°C in hybridization buffer (5X SSPE, 50% formamide, 5X Denhardt's solution, 0.1% SDS, and 0.1 mg/ml denatured salmon sperm DNA). Using ^32^P labeled iNOS or hMnSOD cDNA, the membrane was hybridized at 42°C for 48 h. Using a buffer containing 5X SSC, 0.1% SDS, and 0.05% sodium pyrophosphate, the blot was washed for 15 min at room temperature twice. The last washing step was performed at 65°C in 0.1X SSC, 0.1% SDS, and 0.05% sodium pyrophosphate. The blot was then exposed to Kodak x-ray film at −80°C overnight.

### Animal treatment and tissue isolation

Male mice between the ages of 8–10 wks received one intraperitoneal (i.p.) injection at a dosage of 20 mg/kg ADRIAMYCIN PFS® (Pharmacia & Upjohn, Kalamazoo, MI) while control mice received saline solution. At various time points, mice were anesthetized using Nembutal® sodium solution (65 mg/kg) (Abbott Laboratories, North Chicago, IL). Blood was removed from the left ventricle by cardiac puncture using a 1-ml syringe with a 25G^5^/_8_ needle. Blood was transferred to a Microtainer® Brand Serum Separator Tube (Becton Dickinson and Company, Franklin Lakes, NJ) and allowed to clot for 2 h at room temperature. The serum was obtained by centrifugation at 6000×*g* at room temperature for 5 min. Serum was analyzed immediately upon isolation. The heart tissue was homogenized by polytron in 0.05 M potassium phosphate buffer, pH 7.8, sonicated on ice for three 15 s bursts using 10% output, and frozen for enzymatic assay of SOD.

### Enzymatic SOD activity assay

Protein concentration of homogenate was determined using a colorimetric assay (BioRad Laboratories, Hercules, CA). The SOD activity of each sample was determined by the NBT method as described previously [8]. In order to assay the activity of MnSOD, NaCN (5×10^−3^) was added to the mixture to inhibit copper, zinc superoxide dismutase (CuZnSOD) activity. Enzyme activities (Units/mg protein) are from 6 animals of each genotype.

### Western blot analysis

Protein concentration of homogenate was determined using a colorimetric assay (BioRad Laboratories, Hercules, CA). The homogenate (20 µg) was electrophoresed using SDS-PAGE method and transferred to a nitrocellulose or PVDF membrane. The membrane was washed and then blocked in Blotto [5% milk, 10 mM Tris-HCl, 150 mM NaCl (pH 8.0), and 0.05% Tween 20] for 1 h at room temperature. An affinity purified rabbit anti-MnSOD antibody purchased from Upstate or anti-4-hydroxynonenal (4HNE) (Alexis) antibody was used. After two washings in TBST [10 mM Tris-HCl, 150 mM NaCl (pH 8.0), and 0.05% Tween 20], the blot was incubated with goat anti-rabbit IgG conjugated to horseradish peroxidase from Santa Cruz Biotechnology (Santa Cruz, CA) at a 1∶3000 dilution in Blotto for 1.5 h at room temperature. The blot was washed three times in TBST and once with TBS [10 mM Tris-HCl, and 150 mM NaCl (pH 8.0)]. Protein bands were visualized using the ECL detection system from Amersham Pharmacia Biotech (Buckinghamshire, England). An affinity purified sheep anti-CuZnSOD antibody (1∶3000), purchased from Calbiochem or anti-glyceraldehyde-3-phosphate dehydrogenase (GAPDH) antibody (1∶3000) (Trevigen) was used for a loading control.

### Nitrate measurement

Serum was centrifuged using ULTRAFREE®-0.5 Biomax-30 membrane tubes at 10,000×*g* for 30 min. A final volume of 200 µL including 40 µL of sample, 40 µL of nitrate/nitrite assay buffer, and 10 µL each of nitrate reductase co-factor and nitrate reductase enzyme preparation was incubated, covered at room temperature for 3 h. After the addition of 50 µL each of Greiss Reagent R1 and R2, the samples were incubated uncovered for 10 min at room temperature and the formation of a deep purple azo compound was detected using a spectrophotometer at 540 nm. All reagents were obtained from Cayman Chemical (Ann Arbor, MI).

### Electrophoretic mobility shift assay

Heart tissue was excised and nuclear extracts were isolated as previously described, with the inclusion of 35% glycerol and protease inhibitors (pepstatin, aprotinin, leupeptin) at 1 µg/mL in the extraction buffer [9]. Protein concentration was determined by a colorimetric assay (BioRad Laboratories, USA). Oligonucleotides corresponding to NFκB or p53 were purchased from Santa Cruz as follows, NFκ B: 5′-AGTTGAGGGGACTTTCCCAGGC-3′ and p53: 5′-TACAGAACATGTCTAAGCATGCTGGGG-3′. Each oligonucleotide was radioactively end labeled with [γ-^32^P] ATP (3000 Ci/mmol at 10 mCi/mL) and T4 polynucleotide kinase (New England Biolabs, Beverly, MA). The probes were purified on a 20% native page gel. The gel was exposed to Kodak film and the band corresponding to the double strand was excised. The DNA was eluted overnight at 37°C in 300 µL of Tris/HCl, 1 mM EDTA buffer (pH 7.4). The activity of the labeled probe was counted and stored at −80°C. The final volume of each reaction was 20 µL and included 12 µg of nuclear extract protein. In each reaction, 4 µL of 5-fold binding buffer [20% glycerol, 5 mM MgCl_2_, 2.5 mM EDTA, 5 mM DTT, 50 mM Tris/HCl, pH 7.5, 0.25 mg/mL poly (deoxyinosinic-deoxycytidylic acid)] and 40,000 cpm of labeled probe were used. Samples were incubated at room temperature for 20 min. The reaction was stopped by addition of 10X DNA loading buffer [25 mM Tris-HCl (pH 7.5), 0.02% bromophenol blue, and 4% glycerol]. DNA-protein complexes were separated from unbound probe on a native 6% PAGE gel in 0.5X Tris-borate-EDTA buffer. Gels were vacuum dried and exposed to Kodak film at −80°C.

### Real time PCR

Total tissue RNA, 3 h following treatment with ADR, was isolated with Trizol (1 heart/1 mL) and further purified using the RNeasy Mini, RNA isolation kit (Qiagen). Total RNA was eluted from the RNeasy Mini columns with 60 µL of RNase-free water. Total RNA was reverse transcribed (1 µg) using an RT kit (Clontech Laboratories). Complimentary DNA was obtained using oligo(dT) primers (Clontech Laboratories) and was stored at −80°C. Primers purchased from Invitrogen and used for quantitative real time PCR are listed in the Table 1. The LightCycler Instrument using LightCycler-DNA Master SYBR Green I (Roche) was used with LightCycler 3.5.3 software to analyze results. Final concentrations in each reaction were 2 µg cDNA, 3 mM MgCl_2_, 5 pmol of sense, 5 pmol of antisense, and 1XSYBER Green mix (DNA double-strand specific dye). Standard curves using 0.5, 1, 2, 4, 5, and 6 µg of cDNA were used to quantitate PCR products. Normalization was two-fold, using same amounts of cDNA for each reaction, as well as, analyzation of a housekeeping gene, GAPDH. Samples were analyzed in triplicates using N = 3 for each murine genotype and treatment.

**Table 1:** 

Gene	Annealing Temp., °C	Number of Cycles	Primer Sequence
MnSOD	55	40	5′-GCTTGATAGCCTCCAGCAAC-3′
			3′-GGCCAAGGGAGATGTTACAA-5′
Bcl-xL	58	25	5′-CCGACTCACCAATACCTGCATCTC3′
			3′-CCAGAAGAAACTGAAGCAGAGAGGG-5′
Bax	59	30	5′-AGGATGATTGCTGACGTGGACACG-3′
			3′-AAGATGGTCACTGTCTGCCATGTGG-5′
GAPDH	50	20	5′-TGAAGGTCGGTGTGAACGGATTTGGC-3′
			3′-CATGTAGGCCATGAGGTCCACCAC-5′

### Immunoprecipitation

Immunoprecipitation studies with nuclear extracts from heart homogenate were performed in RIPA buffer [9.1 mM Na_2_HPO_4_/1.7 mM NAH_2_PO_4_/150 mM NaCl (pH 7.4)/1% (v/v) Nonidet P40/0.5% sodium deoxycholate/0.1% SDS/10 mg/mL PMSF/1 µg/mL aprotinin]. Rabbit IgG TruBlot (eBioscience) was used with a rabbit anti-p65 antibody (Santa Cruz) and a monoclonal mouse anti-p53 (Oncogene). Pre-cleared nuclear extract was incubated with anti-p65 overnight at 4°C. Anti-Rabbit IgG beads were added and incubated for 2 h at room temperature. After centrifugation, beads were washed 4X with RIPA buffer (100 µL). Samples were resuspended in sample loading buffer, subjected to SDS/PAGE [12.5% (w/v) gel] and then transferred to nitrocellulose. After transfer samples were analyzed by Western blotting.

### Statistical analysis

Multiple comparisons for each variable were performed using analysis of variance (ANOVA) with the post hoc Fisher's test. When applicable, the unpaired t-test was used to test the significance of each pre-planned comparison. Data was considered statistically significant for *P*≤0.05. All data is presented as mean ± SEM.

## Results

### Manganese superoxide dismutase activity and protein expression is increased in TgM (+/+) and iNOS (−/−)-TgM (+/+) mice

**Figure 1:**
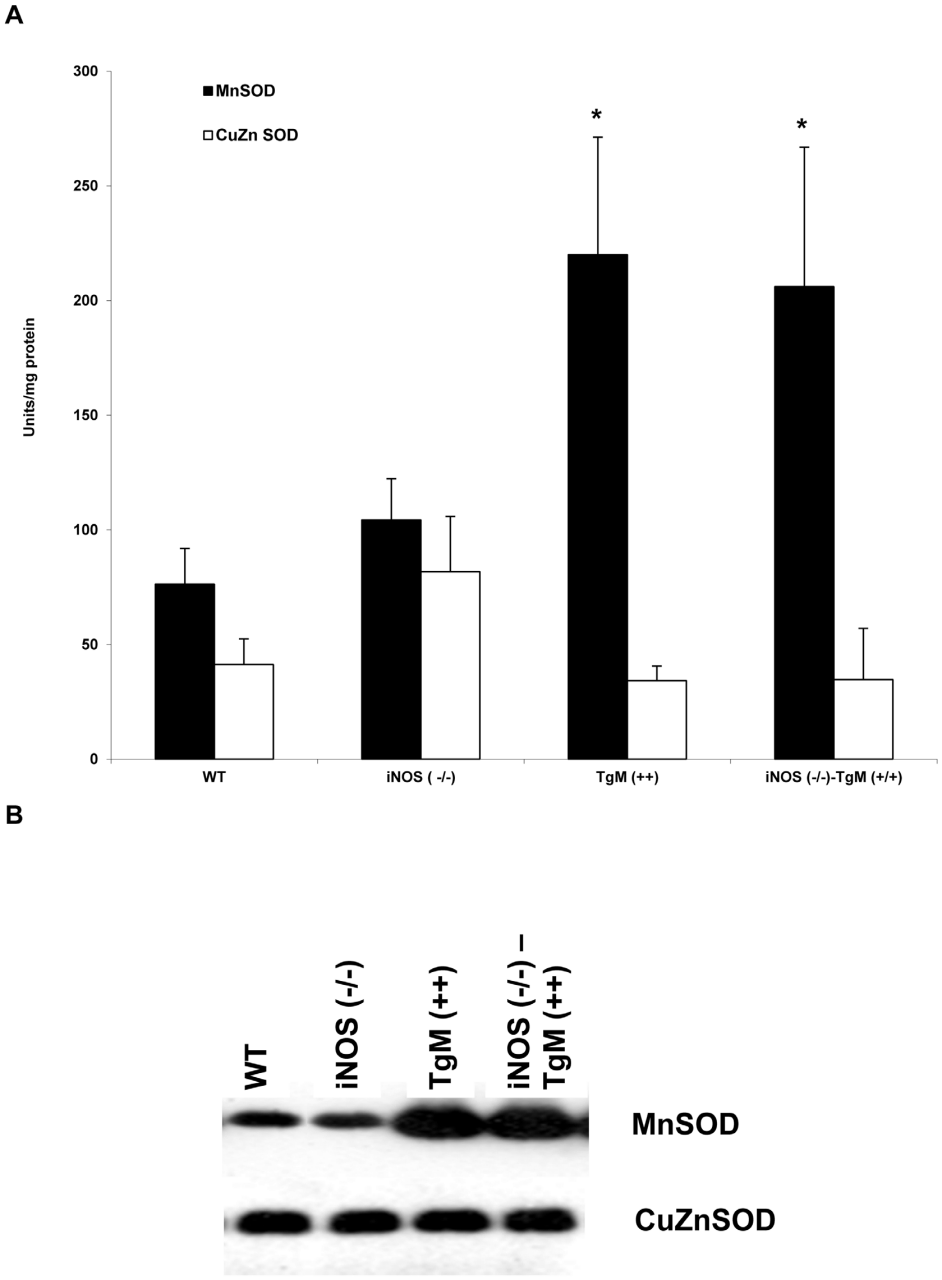
Transgenic mice overexpressing MnSOD have increased enzyme activity and protein expression. (a) Superoxide dismutase activity of heart tissue homogenate in four genotypes: WT, iNOS (−/−), TgM (++), and iNOS (−/−)-TgM (++). Data are expressed as mean ± SEM. *p<0.05 for MnSOD compared to WT and iNOS (−/−). (b) Protein extracts were analyzed by Western blot using an antibody detecting MnSOD. Protein extracts were co-hybridized with an antibody detecting CuZnSOD to assess equal loading of the samples.

Superoxide dismutase enzyme activity was assayed in all four murine models using the method previously described by Oberley *et al.* [8]. Figure 1A shows that levels of MnSOD and CuZnSOD enzyme activity are similar in WT and iNOS (−/−) mice. Mice overexpressing MnSOD have a two-fold increase in enzyme activity with no concurrent change in CuZnSOD activity [Figure 1A, p<0.05 compared to WT and iNOS (−/−)]. Figure 1B demonstrates that overexpression of MnSOD in TgM (+/+) and iNOS (−/−)-TgM (+/+) mice correlates with an increase in enzymatically active protein levels, consistent with activation of enzyme activity (Figure 1A). Copper-zinc SOD protein expression is unchanged.

### Serum nitrate is significantly increased in WT mice

**Figure 2:**
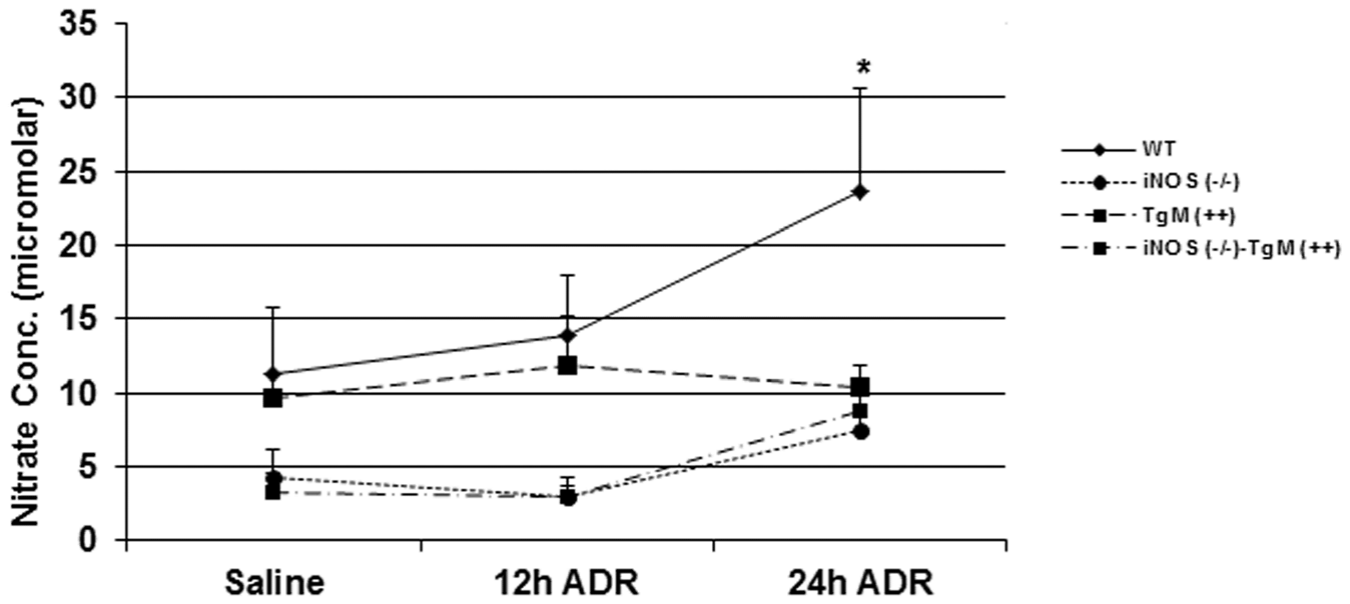
Serum nitrate is significantly increased in WT but not iNOS (−/−), TgM (+/+), or iNOS (−/−)-TgM (+/+) mice following treatment with ADR. Serum nitrate concentration (µM) following time course treatment with ADR (n = 6–8 mice for each time point) was determined. Values are expressed as mean ± SEM. *p<0.0001 versus saline for WT mice.

In order to probe the role of ^•^NO in the mechanism of ADR-induced cardiac injury, serum nitrate concentration was determined following ADR treatment in all four mouse models. Serum nitrate levels increase early following ADR treatment in WT mice and are significantly higher 24 h after ADR treatment (Figure 2, p<0.0001, compared to iNOS (−/−), TgM (+/+) and iNOS (−/−)-TgM (+/+) mice). Previous studies have demonstrated that levels of nitrate in serum are significantly higher in WT mice compared to controls as early as 6 h following ADR treatment [6]. Mice overexpressing MnSOD had similar levels of serum nitrate as WT mice; however, there was no increase in serum nitrate after ADR treatment at 24 h (Figure 2). Basal levels of serum nitrate in both iNOS (−/−) and iNOS (−/−)-TgM (+/+) were lower than WT and TgM (+/+), consistent with the lack of iNOS in both models (Figure 2). There is no statistical difference in serum nitrate in iNOS (−/−) and iNOS (−/−)-TgM (+/+) following treatment with ADR.

### Oxidative injury marker, 4-HNE is elevated at an earlier time point in iNOS (−/−) than WT mice

**Figure 3:**
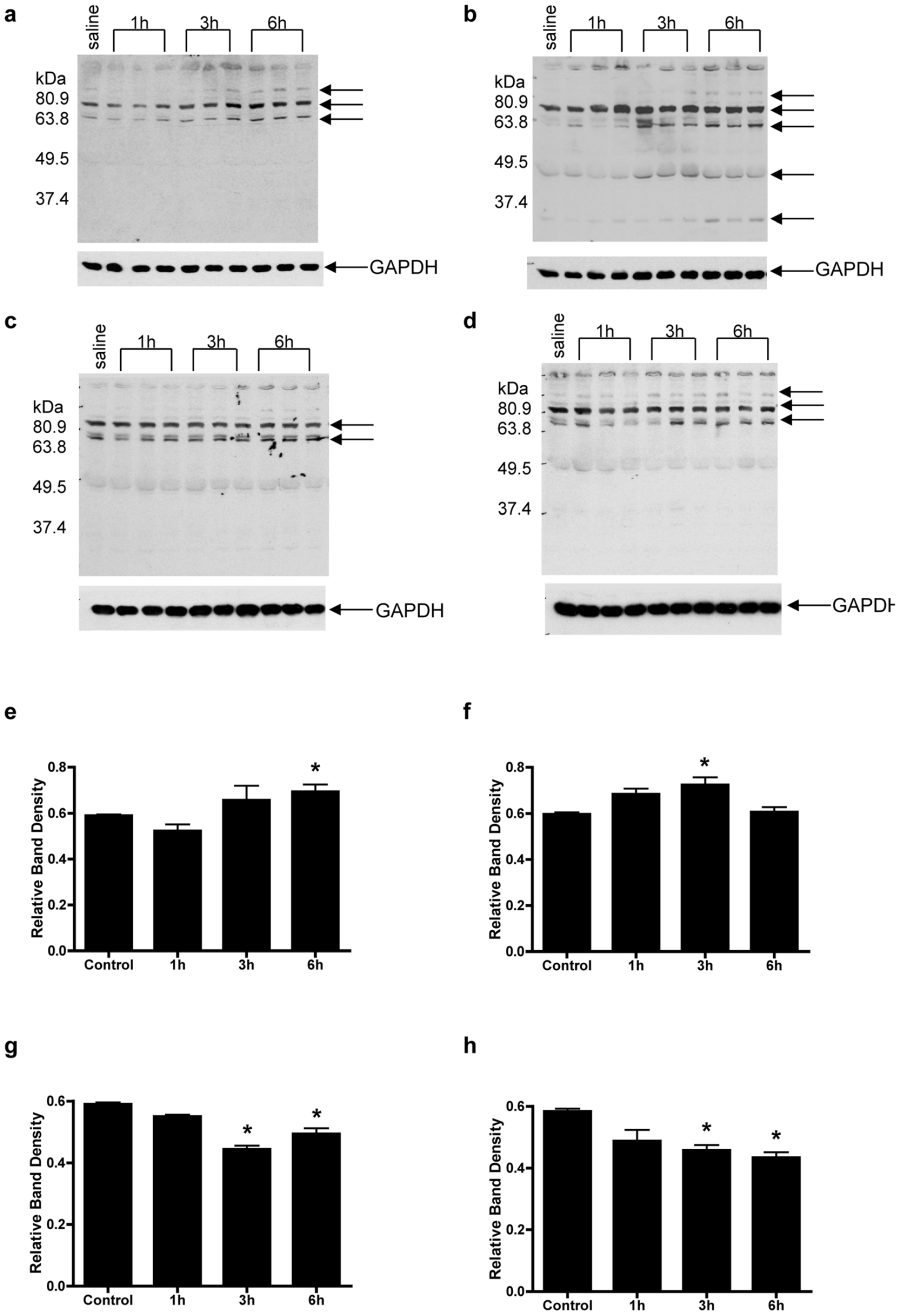
Oxidative injury marker, 4-hydroxynonenal (4-HNE) significantly increases at an earlier time point in iNOS (−/−) mice than WT mice following treatment with ADR. Protein extracts isolated from (a) WT, (b) iNOS (−/−), (c) TgM (++), and (d) iNOS (−/−)-TgM (++) mice were analyzed by Western blot using a 4-HNE antibody after ADR treatment (n = 3 mice for time points 1,3 and 6 h). Membranes were co-hybridized with an antibody detecting GAPDH to assure equal loading of the samples and are quantitated for (e) WT (wildtype), (f) iNOS (−/−), (g) TgM (++), and (h) iNOS (−/−)-TgM (++) mice. *p<0.05 versus saline within each genotype.

We previously reported that mitochondrial oxidative damage precedes nitrative damage in ADR-induced cardiotoxicity, as assessed my electron microscopy [10]. Using Western blot analysis and whole lane densitometry (normalized to GAPDH), Figures 3A and 3E show that WT mice have increased 4-HNE-adducted protein expression beginning at 3 h and statistically significant increases 6 h following ADR treatment (p<0.05). Mice that lack iNOS demonstrated an earlier increase in 4-HNE, at 1 h following ADR treatment, which continued to increase up to 6 h (Figures 3B and 3F; p<0.05). Importantly, Figures 3C, 3D, 3G, and 3H show that both TgM (+/+) and iNOS (−/−)-TgM (+/+) mice have significantly less 4-HNE adducted proteins, respectively, after ADR treatment. The earlier increase in this oxidative parameter implies that iNOS (−/−) mice have an earlier onset of oxidative injury in response to ADR compared to WT mice, which is attenuated by overexpression of MnSOD.

### DNA binding activity of p53 is significantly increased in WT and iNOS (−/−) mice

**Figure 4:**
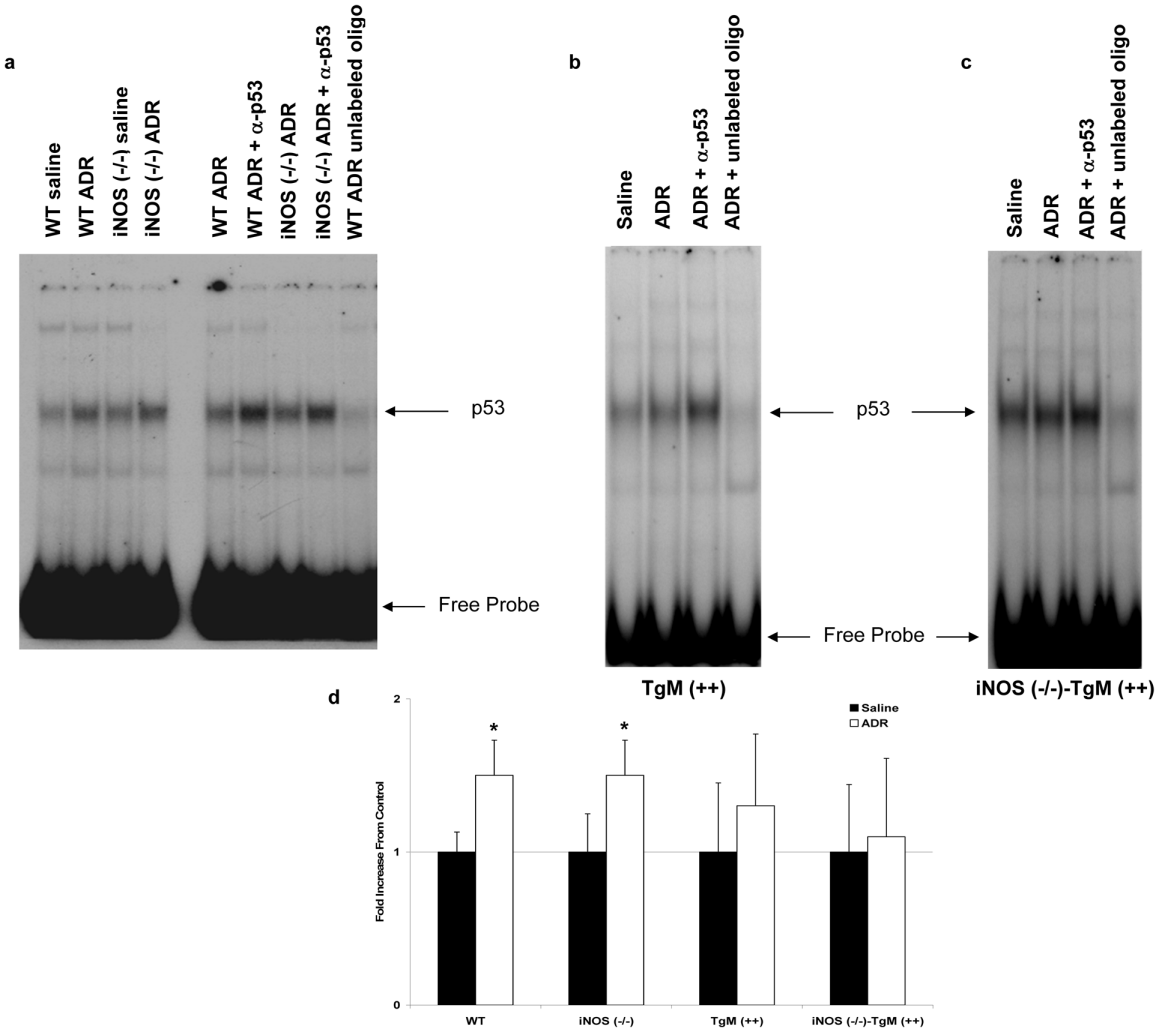
p53 DNA binding activity is significantly increased in WT and mice lacking iNOS. Nuclear extract (12 µg) from heart tissue was isolated, pooled (N = 4–6 animals per lane) from (a) WT and iNOS (−/−), (b) TgM (++), and (c) iNOS (−/−)-TgM (++) mice, and assayed for p53 DNA binding activity. Specificity of DNA binding activity was determined using an anti-p53 antibody, as well as competition with unlabeled p53 oligonucleotide. Densitometric quantitation as fold increase from respective saline control using both individual animals and pooled samples is shown in (d). *p<0.025 versus saline for WT and iNOS (−/−) mice.

The redox-sensitive transcription factor, p53, is known to regulate pro-apoptotic gene products in response to oxidative injury [11]–[13]. Changes in the DNA binding activity of p53 were assessed in all four murine models. Figure 4a shows that WT and iNOS (−/−) mice have significant increases (p<0.025) in p53 DNA binding activity 3 h after ADR treatment. However, iNOS (−/−) mice demonstrated slightly higher basal levels of p53 DNA binding activity than that of WT mice. Transgenic mice treated with ADR have a small, but not significant increase in p53 DNA binding activity as compared to saline controls (Figure 4B). Inducible NOS null mice which overexpress MnSOD, also have a slight, but non-significant increase in p53 DNA binding activity as shown in Figure 4C. Figure 4D illustrates the quantitative increase in p53 DNA binding activity of all four murine models.

### Bax, a target gene of p53 is significantly increased in iNOS (−/−) mice

**Figure 5:**
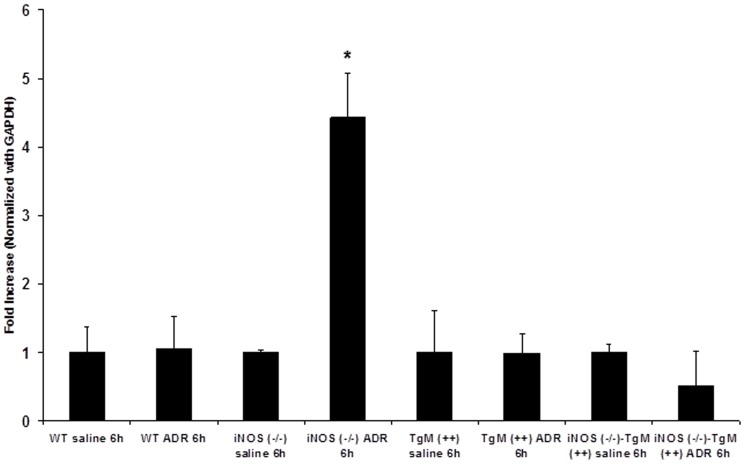
Bax mRNA expression is significantly increased in iNOS (−/−) mice following treatment with ADR. Real time PCR was used to evaluate Bax mRNA expression (6 h) following ADR treatment in WT, iNOS (−/−), TgM (++), and iNOS (−/−)-TgM (++) mice. Data are expressed as fold increase from respective saline control. *p<0.025 versus saline for iNOS (−/−) mice.

The transcription factor p53 is sensitive to oxidative injury and responsible for transcription of the proapoptotic gene, Bax [14]–[18]. The mRNA levels of Bax were assessed in ADR-treated animals. Figure 5 shows that iNOS (−/−) mice had a significant (p<0.025) increase in Bax mRNA after ADR treatment. There were no significant differences in Bax mRNA in response to ADR amongst the three remaining murine models (Figure 5).

### NFκB DNA binding activity is significantly increased in WT mice

**Figure 6:**
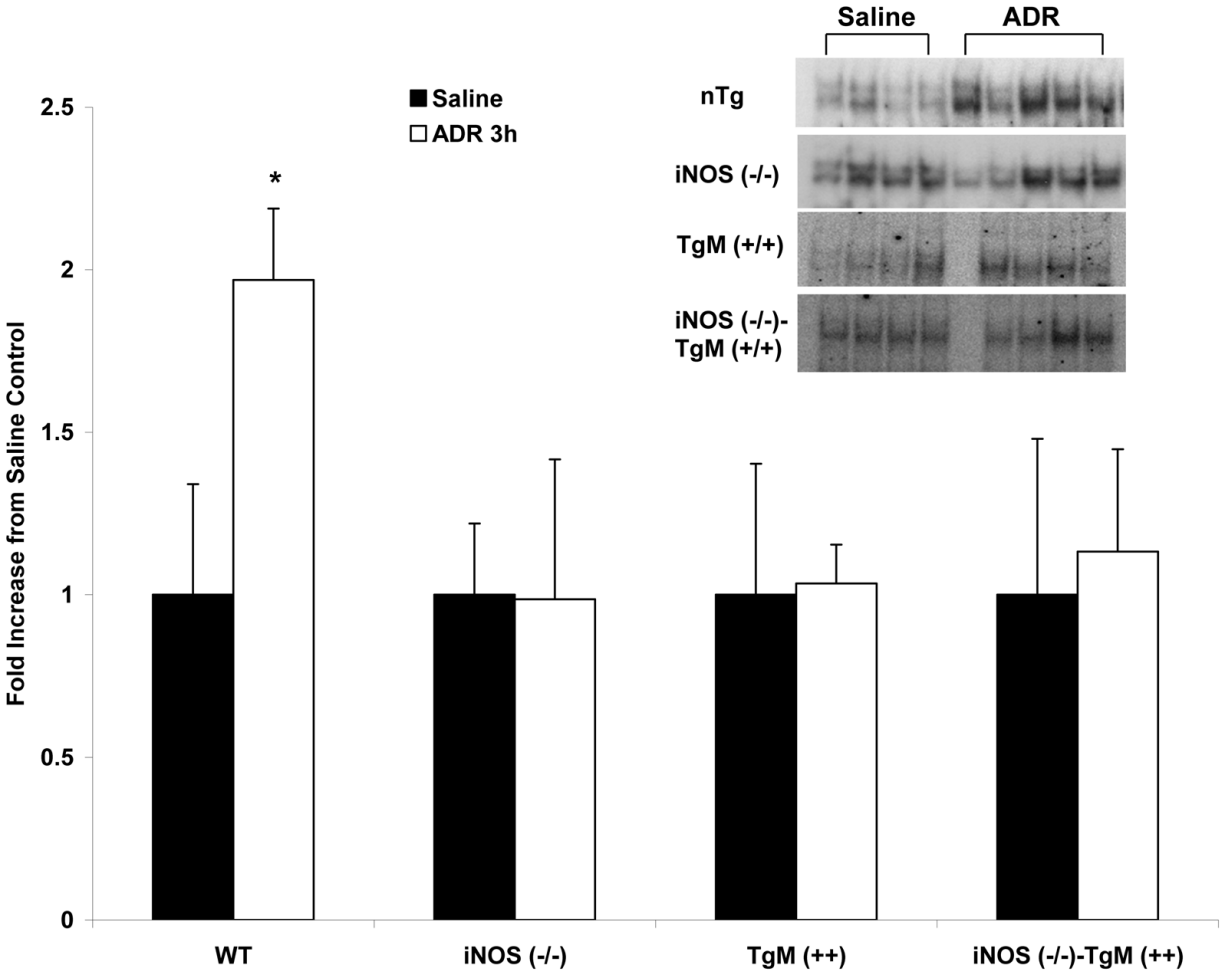
NFκB DNA binding activity is increased in WT mice. Nuclear extract (12 µg) from heart tissue of WT, iNOS (−/−), TgM (++), and iNOS (−/−)-TgM (++) mice was isolated and assayed for NFκB DNA binding activity. Densitometric quantitation as fold increase from respective saline control is shown (with gel shift inset). *p<0.001 versus saline for WT.

To elucidate potential cytoprotective signaling pathways up-regulated in ADR-induced cardiac injury, the DNA binding activity of redox-sensitive transcription factor, NFκB, was determined by EMSA as demonstrated for p53. Wild type animals treated with ADR had a significant increase (p<0.001) in NFκB DNA binding activity at 3 h compared to saline controls (Figure 6). However, mice lacking iNOS demonstrated no significant difference from saline controls 3 h after ADR. Transgenic mice overexpressing MnSOD and mice null for iNOS (−/−)-TgM (+/+) had no change in NFκB response to ADR.

### NFκB target genes, MnSOD and Bcl-xL, do not change following ADR treatment

**Figure 7:**
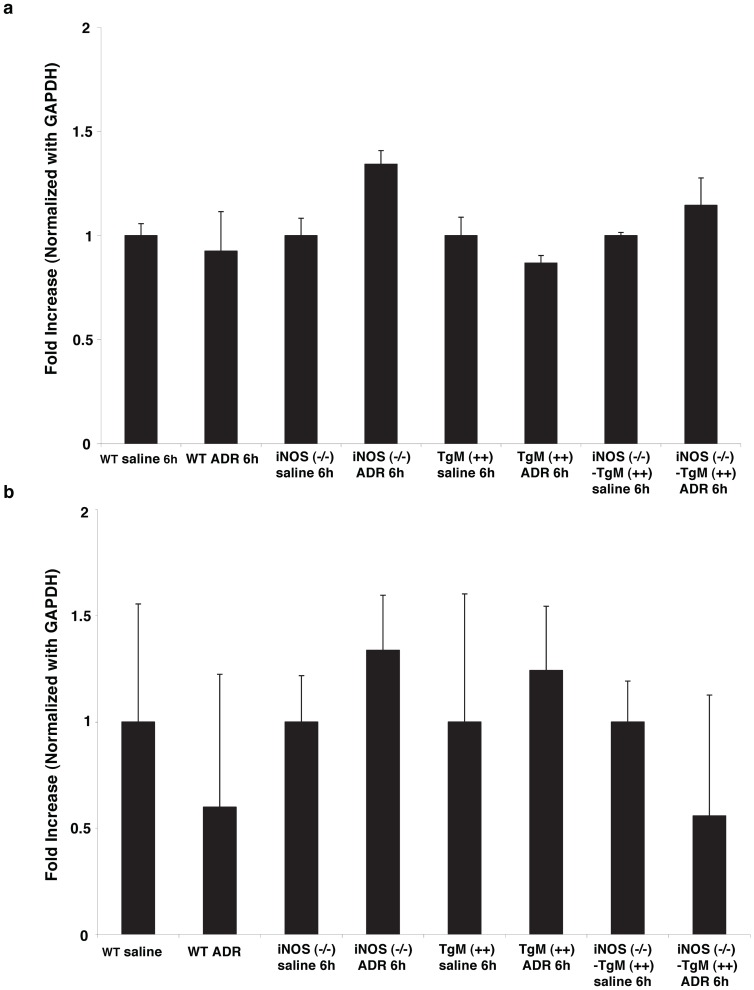
MnSOD and bcl-xL mRNA expression are not changed following treatment with ADR. Real time PCR was used to evaluate (a) MnSOD and (b) Bcl-xL mRNA levels (6 h) following ADR treatment in WT, iNOS (−/−), TgM (++), and iNOS (−/−)-TgM (++) mice.

NFκB is responsible for the transcription of the antioxidant, MnSOD, and anti-apoptotic gene, Bcl-xL [19]–[22]. The mRNA levels of MnSOD and Bcl-xL were assessed by quantitative real time PCR. Figure 7A shows that there were no significant differences in MnSOD mRNA amongst the four murine models in response to ADR. The mRNA levels of Bcl-xL did not change among the four murine models (Figure 7B).

### p53 interacts with p65 in the nucleus after treatment with ADR

**Figure 8:**
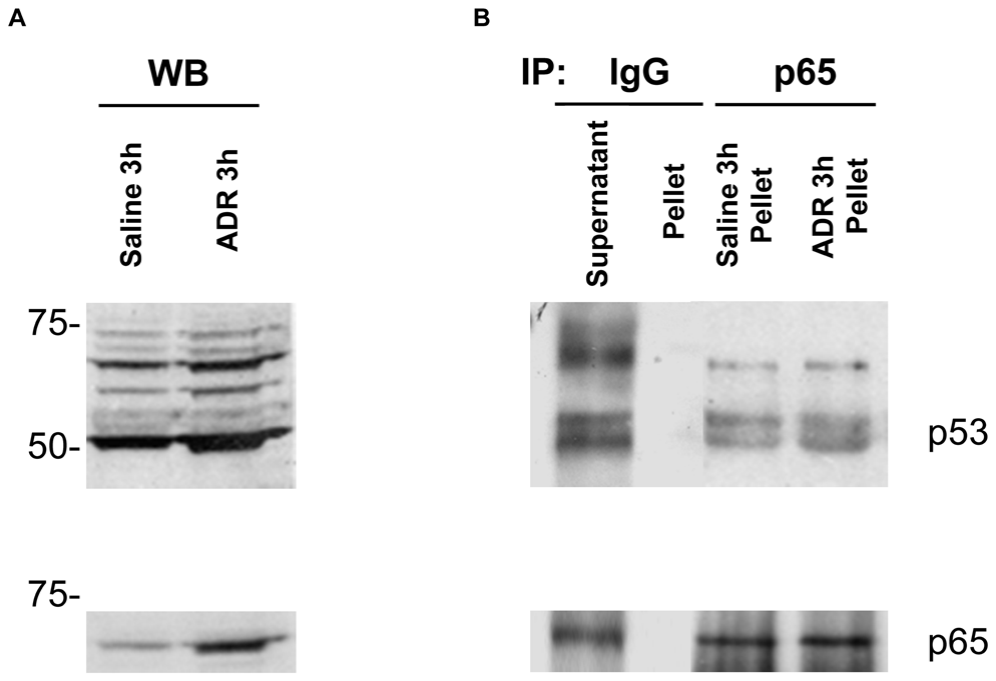
Induction and interaction of p65 with p53 in nuclear extract following treatment with ADR. Expression of p65 increases following treatment with ADR (A). Immunoprecipitation with p65 demonstrating an interaction with p53 in the nucleus after treatment with ADR. Anti-p65 was used to reprobe membrane to verify presence of p65 (B).

In order to determine the role of elevated redox sensitive transcription factors NFκB and p53 in WT mice, we co-immunoprecipitated p65, a prominent member of the NFκB family with an anti-p53 antibody. Using Western blot analysis, Figure 8a demonstrates that p65 protein expression is increased following ADR treatment in WT mice. Importantly, Figure 8b confirms the presence of p65 and the interaction with p53 in the nuclear fraction following ADR treatment.

## Discussion

Adriamycin is a broad spectrum anthracycline antibiotic used for treatment of most solid tumors; however, its clinical usefulness is limited by the potential lethal cardiac injury. The mechanisms by which ADR exerts its anti-tumor effects may differ from the mechanisms resulting in cardiac toxicity [23], [24]. While the significance of ADR-induced production of reactive oxygen species (ROS) in the killing of cancer cells may not be predominately focused on in the literature, the role and concern of ADR-induced ROS in cardiac toxicity is well documented [24]. Advances have been made to further understand the underlying mechanisms associated with both the acute and chronic toxicity of ADR, however cell signaling events, as well as subcellular targets involved are not well understood. Previous studies have demonstrated that ADR affects cardiac specific gene regulation as early as 2 h following administration [25]. The present study focuses on early cell signaling events that precede ADR-induced cardiomyopathy and demonstrates that ADR-induced cardiac injury in wildtype mice leads to co-incidental activation of both cytoprotective and toxic signaling pathways resulting in an interaction of members of the NFκB family with p53. These results underscore the significance of ^•^NO production in the control of cardiac injury.

Using ultrastructural analysis, we have previously shown that ADR has toxic effects on the heart consisting of mitochondrial degradation, loss of cristae, and vacuolization [3], [4], [6]. Additionally, the lack of iNOS in ADR-induced cardiac injury leads to an exacerbation of damage related to an absence of ^•^NO production [3], [6]. Distinctively, iNOS null mice exhibit greater mitochondrial damage over the total area of heart tissue [3]. The increase in mitochondrial injury resulting from the lack of iNOS can be alleviated by overexpression of MnSOD [3], [4], [6]. In contrast, Pacher *et al*. demonstrated that ADR-induced slight improvements in the cardiac function of iNOS (−/−) mice [26]. Variations in measurements of cardiac function exist between ex vivo and in vivo methods and both methods have known limitations. Limitations can include, but aren't limited to, systemic and/or neurohumoral modulation such as CNS and blood volume, which are variables extrinsic of the heart. Importantly, cardiac function measurements ex vivo provide an indication of cardiac status independent of other systolic factors. One other important difference is the background strain of the mice, which can contribute to measured differences between the study performed by Pacher *et al*. and the observations reported herein. The mice used in this study and the previously published results are bred more than 10^th^ generation in B6C3 background compared to C57BL/6 [6]. Measurements of cardiac function are in agreement with the ultrastructural pathology and biochemical data in the same genetic backgrounds and therefore the observed effects of iNOS is documented here.

Formation of 4-hydroxynonenal (4-HNE) adducts can serve as second messenger signal mediators and/or markers of oxidative tissue damage [27], [28]. In an effort to link the production of ROS and RNS production to the exacerbation of injury shown in iNOS (−/−) mice, a time course study was used to analyze for serum nitrate production and 4-HNE protein adducts. Previous reports demonstrate immunohistochemical staining of iNOS and 3-nitrotyrosine following ADR treatment in mice [3], [29]. These results support that iNOS is increased in response to ADR treatment. Additionally, the serum nitrate levels were increased as early as 6 h in WT mice after ADR treatment followed by an increase in nitrotyrosine protein adducts [6]. To extend previous studies, Figure 2 demonstrates that serum nitrates increase up to 24 h following ADR treatment, but only in WT mice. Mice which overexpress MnSOD have basal nitrate levels similar to WT mice, however do not have elevated nitrate levels in response to ADR. Mice lacking iNOS which overexpress MnSOD, have both lower basal levels of serum nitrate and demonstrate no change following ADR treatment. In agreement with our findings, previous studies showed that ADR treatment leads to increases in nitrate levels in heart tissue and serum isolated from rat [11], [30]. Mice lacking iNOS have increased 4-HNE adducts beginning at 1 h following ADR treatment (Figure 3). This increase is statistically significant and earlier than that seen in WT mice at 3 h. These results indicate that levels of oxidative injury due to ADR are higher for iNOS (−/−) mice as compared to WT, and iNOS null mice lacked the ability to increase significant ^•^NO production. In addition, previous studies using immunogold analysis revealed that iNOS (−/−) mice have increased 4-HNE adducts relative to 3-nitrotyrosine levels as compared to WT mice [3], [10]. Overexpression of MnSOD in both transgenic and iNOS (−/−)-TgM (++) mice demonstrate little to no change in 4-HNE levels after treatment with ADR. The attenuation of 4-HNE protein adduct expression in MnSOD overexpressing mice further verifies that O_2_^•−^ mediates ADR-induced tissue damage.

Previous studies have suggested that formation of peroxynitrite occurs following ADR administration [31]. Serum nitrite levels increase in WT mice following ADR treatment (Figure 2) and previous findings demonstrated increases in nitrotyrosine protein adducts [10]. Since it is known that the simultaneous production of ^•^NO and O_2_^•−^ can lead to peroxynitrite formation [32], the data presented in this study further support the findings reported by Mukhopadhyay et al [31]. Peroxynitrite is a potent oxidizing and nitrating species that can give rise to deleterious effects, but can also lead to fatty acid nitration, resulting in formation of adaptive anti-inflammatory mediators in cardiac mitochondria [33], [34]. It is also well known that ^•^NO can easily diffuse into the lipid bilayer, blocking the propagation of lipid peroxyl radicals [35]. Since ^•^NO can react with ADR-derived O_2_^•−^, as well as lipid peroxyl radicals and overexpression of MnSOD attenuates cardiac injury in iNOS deficient mice [6], it is possible that mice lacking iNOS may have excess oxidative stress following ADR treatment.

Transcription factor p53 has been shown to be involved in redox-sensitive signaling under conditions of oxidative stress [13], [14], . Much of the literature has focused on p53 and its relevance to the guardian of the genome [14], [18], [36], [37]. Levels of p53 are known to increase following oxidative challenging conditions causing cellular stress such as UV radiation, hypoxia, heat shock, and cytotoxic drug treatments [12], [13]. Importantly, transcription activity of p53 has been associated with regulation of apoptotic genes in the mitochondria such as Bax, p21, AIF, Fas/APO1, and Noxa [23]. Here we report that the DNA binding activity of p53 is significantly increased in WT and iNOS (−/−) mice 3 h following treatment with ADR (Figure 4A). Increased levels of p53 in WT and iNOS (−/−) mice correlates with the presence of 4-HNE protein adducts as compared to the lack of a change in p53 levels following ADR in mice overexpressing MnSOD.

The redox sensitive transcription factor, NFκB, is increased only in WT mice 3 h following ADR treatment as compared to saline controls (Figure 6). The increase in NFκB DNA binding activity is correlated with the early increases in serum nitrate production found in WT mice, but absent in iNOS (−/−) mice, indicating that ^•^NO or nitrosative stress may mediate a cell signaling response to ADR treatment. However, mice lacking iNOS had no change in NFκB binding activity after ADR treatment. This data in combination with 4-HNE analysis also suggests that ADR-induced oxidative injury, as opposed to nitrosative stress, mediates the signaling response in mice lacking iNOS. This result is consistent with the findings reported by Zingarelli et al. showing that the absence of iNOS enhances myocardial damage in ischemia reperfusion injury (IRI) [38]. Specifically Zingarelli et al. showed that the binding activity of NFκB and AP-1 were significantly reduced in mice lacking iNOS as compared to controls, indicating that iNOS was responsible for a beneficial role in IRI [38]. Transgenic mice and iNOS (−/−) mice overexpressing MnSOD show no change in the binding activity of NFκB after ADR treatment. Manna *et al.* reported a similar result whereby MCF−7 cells overexpressing MnSOD and stimulated with tumor necrosis factor (TNF), a known inducer of NFκB, had no change in NFκB DNA binding activity [39]. Additionally, transgenic mice lacking iNOS, but overexpressing MnSOD had no significant changes in serum nitrate production, or nitrosative stress response to ADR treatment. Downstream gene products regulated by NFκB signaling were unchanged in WT mice (Figure 7). Although, p53 DNA binding activity was increased in WT and mice lacking iNOS, Bax was only increased in iNOS deficient mice. Importantly, p53 was co-immunoprecipitated with p65 in nuclear extracts following treatment with ADR. This data suggests that p53 and NFκB counteract each other in WT mice. This finding suggests that NFκB negatively regulates p53.

These results overall suggest that ^•^NO mediates a cellular response to ADR treatment in WT mice eliciting an increase in binding activity of NFκB. The DNA binding activity of p53 significantly increases in WT and mice lacking iNOS. Additional studies report that p53 represses *SOD2* expression at the promoter level [40]. This result could possibly explain why there is no change in the mRNA expression of MnSOD in WT mice after ADR treatment.

In summary, 1) transgenic and mice lacking iNOS, but overexpressing MnSOD do not have significant increases in p53 binding activity, perhaps due in part to reduced levels of ROS mediated apoptosis signaling leading to the observed protection when challenged with ADR treatment, 2) mice which lack iNOS are unable to produce a high level of ^•^NO production and therefore have an absence of enhanced NFκB binding activity suggesting a role for ^•^NO in NFκB activation, 3) WT mice have increased NFκB and p53 DNA binding activity, but there is no change in downstream gene products, suggesting that interaction of p53 with NFκB inactivates p53 transcription activity, which is further supported by the fact that iNOS (−/−) mice are unable to activate NFκB, but exhibit increased Bax mRNA, a known p53 target gene. Overall, these studies indicate that ^•^NO production, thereby regulating NFκB and p53 pathways, is protective in ADR-induced cardiac tissue signaling. Therapeutic interventions limiting the production of ^•^NO may be clinically detrimental, especially where ADR chemotherapy is involved. Moreover, these results support recent findings where oral delivery of inorganic nitrates is suggested for patients receiving ADR [41]. Further studies are required to demonstrate overall protection provided by ^•^NO/nitrate in ADR-induced cardiac toxicity.
